# Lumbosacral Transitional Vertebrae amongst the Individuals Undergoing Magnetic Resonance Imaging of the Whole Spine in a Tertiary Care Hospital: A Descriptive Cross-sectional Study

**DOI:** 10.31729/jnma.6336

**Published:** 2021-10-31

**Authors:** Subindra Karki, Ramchandra Paudel, Arun Phuyal, Anupam Bhandari

**Affiliations:** 1 Department of Radiodiagnosis and Imaging, Kathmandu University School of Medical Sciences, Dhulikhel Hospital, Kathmandu University Hospital, Dhulikhel, Kavre, Nepal

**Keywords:** *lumbosacral transitional vertebrae*, *magnetic resonance imaging*, *Nepal*

## Abstract

**Introduction:**

Lumbosacral transitional vertebrae is a common congenital anomalous condition of the spine. Recent advances in magnetic resonance imaging have made it possible to acquire images of the whole spine. This study aimed to find out the prevalence of lumbosacral transitional vertebrae amongst the individuals undergoing magnetic resonance imaging of the whole spine in a tertiary care hospital.

**Methods:**

A descriptive cross-sectional study was conducted in 750 patients of all age groups who underwent magnetic resonance imaging of the whole spine in the Department of Radiodiagnosis and Imaging, Kathmandu University School of Medical Sciences from 7^th^ November, 2019 to 6^th^ November, 2020. Convenience sampling technique was used. Ethical approval was taken from the Institutional Review Committee (Reference number 207/19). Data was analysed using Statistical Package for Social Sciences version 22. Point estimate at 95% Confidence Interval was calculated along with frequency and percentage.

**Results:**

Lumbosacral transitional vertebra was seen in 98 (13.10%) (95% Confidence Interval= 10.6115.51) of the total 750 individuals. Out of the 98 patients who had lumbosacral transitional vertebra, 31 (4.10%) had lumbarization of S1 vertebra and 67 (8.94%) had sacralization of L5 vertebra.

**Conclusions:**

Prevalence of lumbosacral transitional vertebrae amongst the individuals undergoing magnetic resonance imaging of the whole spine in our hospital was similar to other study done in similar settings. Lumbosacral transitional vertebrae are a common congenital anomalous condition of the spine that is identified incidentally. Enumeration of vertebrae from the first cervical vertebra using whole spine magnetic resonance imaging can confirm the presence of the lumbosacral transitional vertebrae with much accuracy

## INTRODUCTION

Lumbosacral transitional vertebra (LSTV) is an anomalous vertebra with intermediate morphological characteristics between the sacral and lumbar vertebrae. There can be either sacralization of the lowest lumbar vertebra (L5) or lumbarization of the most superior sacral vertebra (S1).^1^ Radiological imaging modalities like plain radiograph of the lumbosacral spine, Computed Tomography (CT) and Magnetic resonance imaging (MRI) are used to diagnose the condition.

Even though various anatomical landmarks are used for identification of LSTV, they can still have variations in themselves leading to chances of error. Recent advances in MRI have made it possible to acquire images of the whole spine.

This study aimed to find out the prevalence of lumbosacral transitional vertebrae amongst the individuals undergoing magnetic resonance imaging of the whole spine in a tertiary care hospital.

## METHODS

This was a descriptive cross-sectional study conducted in 750 patients of all age groups who underwent MRI of the Whole Spine in the Department of Radiodiagnosis and Imaging, Kathmandu University Dhulikhel Hospital, Dhulikhel, Kavre from 7^th^ November, 2019 to 6^th^ November, 2020. Ethical approval was granted by Kathmandu University School of Medical Sciences Institutional Review Committee (Ref no. 207/19). Patients with prior history of lower spinal surgery were excluded from the study. Subjects with scoliosis, listhesis greater than grade 1, and/or history or MRI findings of spinal trauma, tumor, surgery or infection were excluded.

The sample size was calculated as,

n = Z^2^ × p × q / e^2^

  = (1.96)^2^ × 0.50 × 0.50 / (0.04)^2^

  = 601

where,

n = required minimum sample size
Z = 1.96 at 95% Confidence Interval
p = prevalence taken for maximum sample size, 50%
q = 1 - p
e = margin of error, 4%

Adding 10% non-response rate, the sample size becomes 662. However, a total sample size of 750 was taken. Written informed consent was obtained from each patient. The objectives and protocol of the study was explained in detail. A clinical data proforma was filled up. MRI was performed as per protocol. Images of the spine MRI were acquired, saved and studied.

Based on the assumption that there are 7 cervical and 12 thoracic vertebrae, the vertebrae were counted from the C2 vertebra downwards. The assessment of spine was solely based on the MRI appearances and not in conjunction with other imaging modalities. The lumbosacral transitional vertebra was called sacralized L5 if it followed after L4, and referred to as lumbarized S1 if it followed after L5. In addition to the routine MRI protocol of lumbosacral spine, whole spine screening T2 weighted sagittal imaging was performed. All examinations were performed on a Philips Ingenia 1.5 Tesla MRI scanner.

Data was entered in Microsoft Excel sheet. IBM Statistical Package for the Social Sciences version 22 was used to analyse the data. Descriptive statistics were presented with frequencies and percentages for categorical variables. Point estimate at 95% Confidence Interval was calculated along with frequency and percentage for binary data.

## RESULTS

Lumbosacral transitional vertebrae were seen in 98 (13.10%) (95% Confidence Interval= 10.61-15.51) out of 750 patients. Out of the 98 patients who had lumbosacral transitional vertebrae, 31 (4.10%) had lumbarization of S1 vertebra and 67 (8.94%) had sacralization of L5 vertebra.

Out of a total of 750 patients, 473 (63.06%) patients were males and 277 (36.93%) patients were females. The minimum age was 18 and maximum was 73 years; the mean age was 30.16± 11.28. Table 1 shows prevalence of lumbosacral transitional vertebrae in our study population (Table 1).

**Table 1:** Prevalence of Lumbosacral transitionalal vertebrae in our study population (n= 750).

Type of variation	n (%)
Sacralization of L5	67 (9)
Lumbarization of S1	31 (4.10)

The prevalence of lumbosacral transitional vertebrae has been presented (Table 2).

**Table 2:** Prevalence of lumbosacral transitional vertebrae.

Type of variation	Gender		Total n (%)
	Male n (%)	Female n (%)	
Sacralization of L5	49 (6.53)	18 (2.40)	67 (8.94)
Lumbarization of S1	25 (3.34)	6 (0.8)	31 (4.10)

When the gender prevalence was calculated, out of the 473 male patients, 74 (15.60%) had lumbosacral transitional vertebra. Amongst these 74 males, 49 (66.21%) had sacralization of L5 and only 25 (33.78%) had lumbarization of S1 . Amongst the 277 female patients who were enrolled in this study, 24 (8.70%) had lumbosacral transitional vertebra. 18 (75%) of these 24 females had sacralization of L5 and 6 had lumbarization of S1.

## DISCUSSION

Plain radiograph of the lumbosacral spine, CT and MRI are the imaging modalities that are used for detection of LSTV. Various anatomical landmarks have been used for the numbering of the vertebrae and detection of the transitional vertebra. Because these anatomical landmarks have variations in themselves, the detection of LSTV by these modalities are prone to error. On sagittal MRI, when the vertebrae are counted from the first cervical vertebra, considering 7 cervical and 12 dorsal vertebrae, the diagnosis of LSTV can be made with more accuracy than with any other modalities.

Although patients with low backache often do not have cervicodorsal imaging, with the invention of 1.5T MRI, screening of the whole spine is routinely performed considering the prevalence of transitional vertebrae and anomalous vertebral number. Screening MR imaging of the whole spine allows visualization of the area of interest and also identifies vertebral variants that can be potentially overlooked and might obscure the accurate enumeration.

The prevalence of lumbosacral transitional vertebra in our study was 13.10%. Our study findings are comparable with those of other authors who have quoted the prevalence rate ranging from 2-30%.^1,3,5,8,11,12^

In our study, the prevalence of LSTV was higher in males as compared with females. Similar findings have also been reported in several other studies.^7,13^ In a study by Hahn PY, et al,^5^ the authors observed a 20 % prevalence of numerical variation, with 14.5% of all patients having lumbarization of S1, 5.3% having sacralization of L5, and one patient (0.13%) having three lumbar vertebra. Eighty percent of patients in their study had five lumbar vertebrae. Similarly, two-thirds of the patients with sacralized lumbar vertebrae were female, while two-thirds of patients with lumbarized S1 vertebrae were male.^5^ However, in our study 13.10% patients who had lumbosacral transitional vertebra, 4.10% had lumbarization of S1 vertebra and 67(8.94%) had sacralization of L5 vertebra which were predominantly seen in male.

There are also some limitations of our study. Numeric abnormalities in the cervical and thoracic vertebrae may create difficulty in diagnosing LSTV. Complex developmental anomalies of the spine (failure of formation and failure of segmentation) can lead to misdiagnosis.

## CONCLUSIONS

The prevalence of lumbosacral transitional vertebrae amongst the individuals undergoing magnetic resonance imaging of the whole spine in our hospital was similar to other study done in similar setting. LSTV is a common congenital anomalous condition of the spine that is identified incidentally. Plain radiograph of the lumbosacral spine, CT and MRI are the imaging modalities that can detect this condition. Various anatomical landmarks are used for the numbering of the vertebrae and detection of the transitional vertebra. Because these anatomical landmarks have variations in themselves, the detection of LSTV by these modalities are prone to error. Enumeration of vertebrae from the first cervical vertebra using whole spine MRI can confirm the presence of the lumbosacral transitional vertebrae with much accuracy.
